# Effectiveness of oral health promotion in children and adolescents through behaviour change interventions: A scoping review

**DOI:** 10.1371/journal.pone.0316702

**Published:** 2025-01-10

**Authors:** Fathima Peerbhay, Robert Mash, Saadika Khan

**Affiliations:** 1 Division of Paediatric Dentistry, Department of Orthodontics, Faculty of Dentistry, University of the Western Cape, Cape Town, South Africa; 2 Department of Family and Emergency Medicine, Faculty of Medicine and Health Sciences, Stellenbosch University, Cape Town, South Africa; 3 Department of Prosthodontics, Faculty of Dentistry, University of the Western Cape, Cape Town, South Africa; Universidad Privada San Juan Bautista, PERU

## Abstract

**Objective:**

To explore the interventions for change in oral health behaviour that are effective in improving oral health behaviours in 8 to 18-year-old children during oral health promotion.

**Methods:**

The Joanna Briggs Institute framework of evidence synthesis for conducting a scoping review was implemented for the methodology. Included studies related to the objective, measured clinical or non-clinical outcomes, were in English, 2011–2023, and were experimental, observational or reviews. PUBMED, Science-Direct, Scopus and Sabinet were systematically searched with predetermined search strings. Studies were selected by appraisal of the title, abstract and full text. Data were extracted using a standardised template and the key questions were addressed via a qualitative analysis.

**Results:**

Searches yielded 407 articles from electronic databases. Of these, 290 articles were excluded, and 47 full-text studies were assessed for eligibility, with 23 studies and two systematic reviews finalised for inclusion. In addition, a PEARL search was conducted from the reference lists of other studies. Most studies (91.3%) focused on educating children directly; 8.7% indirectly influenced parents, guardians, and teachers. Interventions focused largely on traditional oral health education presented in diverse forms and via different platforms. Studies differentiated clinical outcomes (indices) from non-clinical outcomes (knowledge, behaviour). All included RCTs were of different quality regarding selection, performance and detection bias. But all studies indicated a low risk of bias in attrition and Reporting bias. Seventeen of the 25 studies (68%) were not based on any behaviour change theory.

**Conclusions:**

Oral health interventions based on motivational interviewing and the social cognitive theory have been shown to be to be effective. Interventions could also include practical tooth brushing activities, gamification, audio-visual components, as well as reinforcement and repetition in the longer term. Future oral health promotion in children should be designed to include these elements. There is a need for higher quality studies in this field, with future research being urged to provide detailed intervention descriptions and incorporate longer follow-up periods.

## Introduction

Globally, oral diseases continue to present a public health challenge, with the greatest burden of disease in lower socio-economic communities [1, 2]. Oral diseases are one of the most prevalent conditions in low- and middle-income countries [1, 3, 4], with untreated dental caries of deciduous teeth affecting approximately 514 million children [1]. The global prevalence of untreated dental caries in permanent teeth is 29%, affecting more than 2 billion individuals [1].

The risk factors that contribute to dental caries include poor oral hygiene practices, cariogenic diets, limited exposure to fluoride, lack of access to oral healthcare and oral health promotion (OHP), and patients’ low socio-economic status [4, 5]. Oral health promotion interventions that address these risk factors assist in improving oral health and consequently reduce the levels of oral disease [5, 6]. Oral health promotion is a structured effort to achieve oral health goals by creating supportive policies, environments, and community engagement while also enhancing personal skills and adapting health service [6].

Oral health interventions (OHIs) are classified into three types, and they can assist individuals achieve good oral health [4]:

a) *Individual OHIs* refer to oral hygiene practices such as tooth brushing with a fluoride toothpaste and dietary changes [4].
b) Community oral health education (OHE) and water fluoridation fall into the category of *community OHIs*.
c) *Professional OHIs* refer to early identification and appropriate management of oral disease, dietary counselling, and professional fluoride application [4, 5, 7].

Oral health interventions whether on an individual, community or professional level, are essentially all directed towards prompting a change in oral health behaviour to improve the patients’ oral health [8]. The primary behaviours that contribute to improving oral health include tooth brushing with a fluoridated toothpaste and reducing the consumption of sugary foods and drinks [4, 7]. Since dental health behaviour becomes established around the age of 15 years, it is prudent that OHP be instituted in early school grades among children and adolescents [9].

Oral health promotion to prevent dental caries in school-aged children should include the following (A-grade evidence) recommendations [10]. Children should brush their teeth twice a day with a fluoridated toothpaste, have fluoride varnish applied professionally at intervals of three to six months if at high risk, have resin-based fissure sealants placed on permanent teeth, and limit their daily consumption of sugar-containing foods and drinks [4, 10, 11].

Creating behaviour change interventions is more effective when based on a theoretical foundation [12]. This approach targets the root causes of behaviour and helps advance theoretical frameworks in various contexts, demographics, and behaviours [12]. In order for behaviour change interventions to be effective, they need to “target a determinant that predicts behaviour; be able to change that determinant; be translated into a practical application in a way that preserves the parameters for effectiveness and fits with the target population, culture, and context” [[13] p.303].

One type of health behaviour intervention is health education [14]. Health education can be defined as “the communication of information concerning the underlying social, economic and environmental conditions impacting on health, as well as individual risk factors and risk behaviours, and use of the health care system” [[14] p.12]. An OHI that is provided in an educational context and that combines OHE and preventative oral care can reduce caries in children’s permanent teeth [14, 15]. Traditional OHE that focuses on the provision of information in schools, including primary schools, remains the primary focus of OHP programmes, even though it yields a relatively low level of effectiveness in the long term [12, 16, 17]. Traditional OHE does in fact improve oral health literacy, yet it is not an effective approach when used in isolation [18]. Although traditional OHE improves oral health knowledge (OHK) in the short term, it unfortunately only contributes to a transient improvement in oral health behaviour [19]. The effect that traditional OHE has on clinical outcomes such as plaque levels appears to be conflicting [16, 17]. Several studies, however, confirm that OHE does not produce any long-term sustained effects on oral health [12, 16, 17]. Hence, there is a need to consider alternative approaches [17].

In addition, the design of OHIs for school-aged children has lagged far behind behavioural science theory [20]. The OHP approaches that were found to be effective in both adults and adolescents were based on psychological behaviour change models [21, 22]. Therefore, there is a need to explore alternative approaches to modifying oral health behaviour in children in order to address their current high prevalence of dental caries [20].

Few systematic reviews have been published on OHIs [23–25]. The findings of these systematic reviews are conflicting [23–25]. Two of the systematic reviews reported that there is moderate evidence that behavioural interventions are effective in promoting oral health in the short term, but the studies were of poor quality with a high heterogeneity [23, 24]. The systematic review conducted in primary schools reported that there was limited evidence on the effectiveness of OHIs [25]. There is a need for more high-quality studies to be conducted using theory when developing interventions aimed at altering oral-health-related behaviours in children and their parents [25]. Most of the studies in the systematic reviews were focused on OHIs in adolescents but there is a scarcity of information on the evidence of OHIs in pre-adolescents [25].

The aim of this scoping review (ScR) was to explore the types of interventions that are effective in improving oral health behaviours in 8- to 18-year-old children.

## Methods

### Study design

This ScR was based on the steps described in the Joanna Briggs Institute (JBI) framework of evidence synthesis [26] and the Preferred Reporting Items for Systematic Reviews and Meta Analyses (PRISMA) [27–29].

### Review questions

The main research question was as follows:

Which interventions for changing oral health behaviour are effective in improving the oral health behaviours in 8- to 18-year-old children during oral health promotion.

The review also addressed the following sub-questions:

1. What types of interventions for changing oral health behaviour are used during oral health promotion?
2. Which of these interventions are effective in changing oral health behaviour or the outcomes?
3. Which behaviour change or psychological theories underlie effective interventions for modifying oral health behaviour in children (8–18 years)?

### Identification of relevant studies

#### Eligibility criteria

The inclusion and exclusion criteria were as follows:

- Study population: Children aged 8–18 years. Independent tooth brushing is recommended in children from 8-years-old onwards as this is when hand-eye coordination becomes more established [30]. Studies of children with special needs were excluded.
- Nature of the intervention: Changing oral health behaviour.
- Outcome variables: Oral health outcomes such as dental caries, plaque, gingivitis, and periodontitis. Secondary oral health outcomes were oral-health-related behaviour, oral-health-related quality of life, health beliefs and attitudes, and self-perceived oral health.
- Time period: 2011–2023.
- Cultural and linguistic range: English.
- Types of study design: These included randomised controlled trials and other experimental studies, observational studies, qualitative studies, and systematic and ScRs.

#### Information sources

Relevant published studies were identified using a carefully structured search strategy in the following electronic databases: PUBMED, Science-Direct, Scopus, and Sabinet. These databases are some of the most commonly used in Dentistry, and Sabinet was added to include publications in South Africa and Africa. Only articles published during the time period 2011–2023 were included in the study. In addition, a PEARL search was also performed by examining the reference lists of other studies.

#### Search terms

The search string was created using keywords and Medical Subject Headings (MeSH), and terms were combined using Boolean operators as shown below. This search string was then used to search within the specific databases.

[[“Dental Care for Children” [Mesh] OR (Oral AND Health AND children)] **AND** [(“Health Promotion” [Mesh] **OR** “Risk Reduction Behaviour” [Mesh]) **OR** Health behaviour].

The search string was modified slightly for the search engine requirements of each database, and the number of articles that was retrieved was recorded.

#### Selection of studies

The researchers (FP and SK) used a three-step screening approach. Initially, the titles were screened, then abstracts were evaluated, and thereafter, full-text articles were assessed independently for inclusion, guided by a study eligibility form created for this purpose. Any disagreements were resolved by discussion between the two reviewers. If agreement was still not reached, a third reviewer (RM) adjudicated the discussion. The reviewers recorded the number of included and excluded articles and the reasons for exclusion.

#### Extraction of data

Data was extracted independently by each reviewer (FP and SK) and collated in tabular form using Excel spreadsheets. Data extraction was completed using a form developed by the reviewers, and the data from each full-text article was extracted in a standardised manner and summarised in a template. The fields of extracted information included the author, country, study design, participant demographics, intervention, psychological theory/framework, outcomes, and conclusion.

#### Data analysis

The characteristics of the included studies were analysed descriptively using frequencies and percentages for categorical data. The extracted data that addressed the review questions was interpreted qualitatively, and the findings were synthesised in a narrative review. A risk of bias (RoB) assessment was also conducted for any clinical trials. This assessment evaluated randomisation processes, allocation concealment, blinding, completeness of outcome data, reporting of all outcomes, and any other sources of bias. In the RoB assessment, studies were categorised to indicate a low, high, or unclear RoB [29].

### Ethical considerations

Although ethical approval is not usually required for ScRs, the study protocol received institutional ethics clearance (HREC2-2020-13351) as part of a larger study.

## Results

The results for this study are presented in three specific sections:

- Search results
- Types and effectiveness of OHIs used during OHP with children
- Psychological theories underpinning effective OHIs in children

### Search results

**Fig 1:**
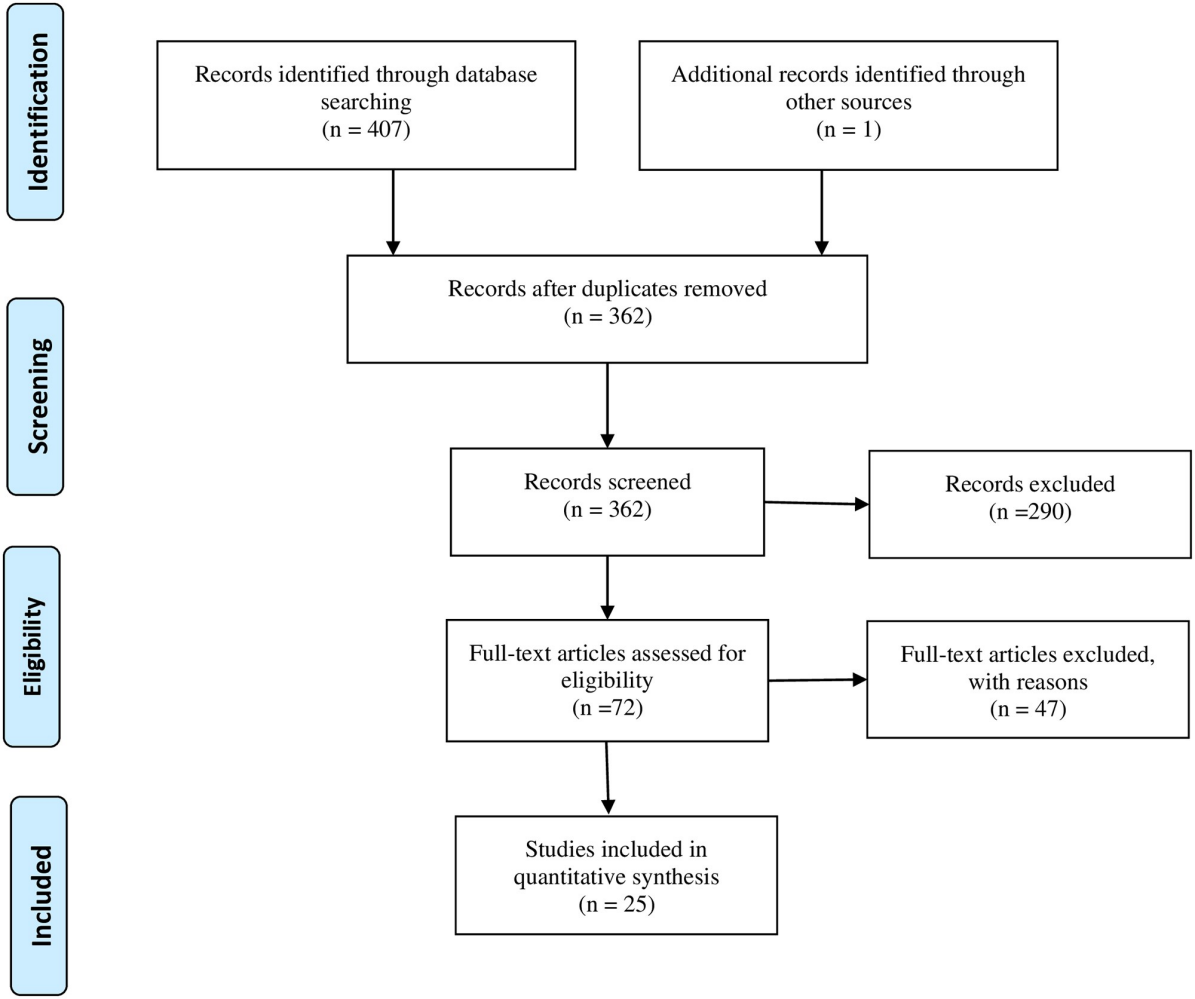
PRISMA flow diagram.

The searches identified 407 articles (Fig 1), and following the three-step screening process, 25 studies were included in the review. The details of the included studies are presented in Table 1 and their characteristics are summarised in Table 2.

**Table 1:** Studies included in the scoping review.

Author	Year	Country	Study design	Participant demographics	Intervention	Psychological theory/framework	Outcomes	Conclusion
1. Bica et al. [31]	2015	Portugal	Before-and-after study of paired groups	Children: 11−16 yrs. N = 200	Four sessions of health education in the classroom were held a month apart. The first two sessions were theoretical and involved oral health, hygiene, and food health. Each session lasted 45 minutes. The remaining two sessions were practical and were based on models and self with tooth brushing. Each lasted 90 minutes. Audio-visual materials, demonstrations, and educational games were included, and pamphlets were provided.	None	Oral health behaviour Oral health status: Plaque Index Gingival Index Dental Caries Index	Reduced percentage of decayed teeth (51% to 39%); improved eating habits; higher percentage of teeth without plaque; more tooth brushing, and reduced risk of future oral problems were noted.
2. Halonen et al. [32]	2013	Finland	Before-and-after study of unpaired groups	Children: 7–12 yrs. N = 1185 (before) N = +1293 (after)	An oral health promotion campaign was conducted over one year and involved all schoolchildren in a municipality. Dental education was given to all children, and some children were selected for tooth-brushing school. Parents also attended the tooth-brushing school.	None	Oral health behaviour: Tooth-brushing frequency Use of fluoride toothpaste Use of dental floss Use of fluoride tablets	Tooth-brushing frequency increased (61% to 65%). Flossing and use of fluoride tablets also increased, but the use of fluoride toothpaste did not. The effect was more evident in younger children (7–10 years) and with parental reminders. Girls adhered to tooth brushing more than boys.
3. Setiawati et al. [33]	2020	Indonesia	Quasi-experimental study	Children: 7–9 yrs. Intervention group: 66 children 66 mothers 20 teachers Control group: 54 children 54 mothers 10 teachers	Pre-test and post-test questionnaires were used to gather information on the behaviour and knowledge of teachers and mothers. The children in the control group received theoretical instruction from their teachers and mothers. Children in the intervention group received a 16-surface tooth-brushing programme, and their Plaque Index was measured a month later.	None	Oral heath behaviour Oral health knowledge Oral health status: Plaque Index	There was an increase in teachers’ oral health knowledge (16.7%), and oral health behaviour (20%) and in mothers’ oral health knowledge (16.7%) and oral health behaviour (20%). The children’s Plaque Index decreased by 47%.
4. Kapoor et al. [34]	2019	India	Quasi-experimental study	Children: 6–10 yrs. n = 50 in each group with parents N = 100 Control: Traditional oral health education; verbal advice and one reminder	Children and their parents were split into two groups. The first group received traditional oral health education from a single calibrated examiner. The parents of this group received one telephone call before the end of the three-month period informing them of their child’s subsequent evaluation. The second group received a 30-minute motivational interviewing session and watched a video on methods of preventing tooth decay. Parents of this group received telephone calls once a month for a year to reinforce the behavioural change.	Motivational interviewing	Oral health status: Dental caries	A single motivational interviewing intervention changed the reported oral health behaviours better than the traditional approach. There were no new caries and initial caries were arrested.
5. Harikiran et al. [35]	2017	India	Before-and-after study with paired groups	Children: 12−13 yrs. N = 45	A card game called ‘32 Warriors’ was developed and included dental health information that was appropriate for children. The card game was designed to empower children to take good care of their oral health. A 32-item, closed-ended questionnaire with a pre-post design evaluated the children’s understanding of oral health, their attitudes towards oral heath, and their opinions of the game.	No psychological theory reported. Constructivism approach used.	OH knowledge OH attitude	The ‘32 Warriors’ card game appeals to children and improves their attitudes and knowledge of dental health. In addition, it has the potential to enhance both oral and overall health.
6. Haleem et al. [36, 37]	2012 and 2016	Pakistan	Randomised controlled trial	Children:10–11 yrs. N = 1517	Five groups of students from 40 schools were involved: three received oral health education from dentists, teachers, or peers, one was self-learning, and one served as a control group. Oral health education for the dentist-led, teacher-led, and peer-led groups was given initially in January 2004, with monthly reinforcement from September 2004 to February 2005. Four evaluations were conducted: immediately after the first session, six months later, and then six months and a year after reinforcement. The self-learning and control groups were surveyed at baseline and after two years. Clinical examinations were conducted by a calibrated dentist.	Social Cognitive Theory	Oral health status: Plaque Index Gingival Index Oral health knowledgeOral health behaviour	The dentist-led, teacher-led and peer-led strategies of oral health education are equally effective in improving the oral health knowledge and oral health status of adolescents. Repetition and reinforcement play a key role in school-based oral health education, irrespective of educators. Trained teachers and peers can play a complementary role in repeated and reinforced oral health education.
7. Vangipuram et al. [38]	2016	India	Randomised controlled trial	Children: 12−15 yrs. N = 450 Peer group: 150 Dentist group: 150 Control: 150	.. Oral health education was provided by both peers and dentists to one group each, using methods such as PowerPoint presentations, traditional lectures, visual aids (charts, posters), informational booklets, and practical tooth-brushing demonstrations The control group received no intervention. A calibrated examiner conducted clinical evaluations, assessing plaque and gingival indices	None reported	Oral health status: Plaque Index Gingival Index Oral health knowledgeOral health attitudeOral health practices	Educational interventions led by peers and dentists were equally effective. Oral health knowledge, oral health practice and oral health status improved. The intervention led by the peers was more effective in improving oral health knowledge and oral health practice.
8. Åstrøm et al. [39]	2012	Tanzania	Before-and-after study with paired groups	Mean = 13.8 yrs. N = 1 306	Primary school students participated in a combined atraumatic restorative treatment / oral health education programme. After six months, a follow-up study was conducted to evaluate changes in their oral-health-related knowledge, attitudes, and behaviours. Among these students, 221 had both baseline and follow-up interviews and received atraumatic restorative treatment / oral health education, while 1 085 students received only oral health education. Clinical examinations were conducted by a calibrated dentist.	Theory of Planned Behaviour	Oral health attitude Oral health behaviour Oral health practices	School-based atraumatic restorative treatment and oral health education programmes improved children’s oral health attitudes, oral health behaviours, and oral health practices. Atraumatic restorative treatment / oral health education programmes had no effect on sugar consumption.
9. D’Cruz et al. [40]	2013	India	Randomised controlled trial	Age: 13–15 yrs. N = 600	Three schools were randomly divided into Experimental I, Experimental II, and Control groups. A 20-item questionnaire was administered, and clinical examinations were conducted to assess oral hygiene knowledge and practices. Experimental Group I received oral health education through PowerPoint presentations, while Experimental Group II received the same plus tooth-brushing demonstrations. The Control group received no intervention. Reinforcement sessions were held for the experimental groups at three and six months. After nine months, a follow-up questionnaire was administered, and clinical examinations were conducted. Clinical examinations were conducted by two calibrated dentists	None Reported	Oral health knowledge Oral health practices Oral health status: Plaque Index Gingival health	Active involvement of school children and reinforcement of oral health education improved oral health knowledge, oral health practice, gingival health, and Plaque Index.
10. Eden et al. [41]	2019	Turkey	Randomised controlled trial	Age: 9 yrs. N = 1 053	This clinical trial compared two oral health education programmes. One group received oral health education from dentists in a classroom lecture, while the other group received a similar lecture from schoolteachers in addition to ongoing support materials for the academic year. The study assessed oral health knowledge and behaviour through questionnaires at baseline, one month, and six months. Clinical examination was conducted by calibrated examiners.	None reported	Oral health knowledge Oral health behaviour Oral health status: • Plaque Index	Both oral health education programmes generated an improvement in oral health knowledge, oral health behaviour (tooth brushing), and plaque control in the short term.
11. Angelopoulou et al. [42]	2015	Greece	Randomised controlled trial	Age: 10 yrs. N = 184 (Experiential: 84; Traditional: 100)	Data on oral health knowledge, attitude, and behaviour was gathered through questionnaires, while clinical examinations assessed dental plaque, gingivitis, and caries. Evaluations using questionnaires and clinical examinations took place at baseline, 6 months, and 18 months. The teacher delivered the oral health educational programme in the classroom using experiential learning. The dentists delivered the traditional lecture on oral health education, and this served as the control. Two calibrated paediatric dentists conducted the clinical examinations.	None reported	Oral health knowledge Oral health behaviour and attitude Oral health status: Plaque Index Gingival Index	Experiential programme was more successful than a traditional lecture in improving oral health.
12. Blake et al. [43]	2015	United Kingdom	Before-and-after study paired groups	Age: 10–11 yrs. N = 150	Children were involved in a 60-minute classroom-based instructional session on oral health led by a dental care professional. They were given printed materials about dental health to read at home. The primary data collection involved the use of a questionnaire that assessed the children’s knowledge of oral health and their self-reported oral health behaviours. Assessments were done before the instructional session, immediately after, and six weeks later.	Theory of planned behaviour	Oral health knowledge Oral health behaviour (tooth brushing, diet)	School-based preventative oral health education can generate short-term improvements in children’s oral health knowledge and oral health behaviours.
13. Niranjan and Knight [44]	2017	South Africa	Randomised controlled trial	Age: 10–14 yrs. Median age: 11 yrs. N = 337 (intervention) N = 337 (control)	In the intervention group, an oral health education video made by Colgate^®^ was shown. This screening was repeated at three-month intervals over a period of nine months. The same Colgate^®^ oral health education video was shown to students in the control schools but only once at the beginning of the trial.	None reported	Oral health knowledge Oral health practices	Repeated oral health education significantly improved oral health knowledge and oral health behaviours.
14. Toratti et al. [45]	2020	Finland	Before-and-after study of paired groups	Age: 13–15 yrs. N = 112: Phase I N = 66: Phase II	A computer-assisted intervention with personalised feedback was carried out to determine its effect on oral health behaviours. The program included 19 questions about oral health practices and provided tailored guidance for better oral health. The intervention was repeated after four weeks.	None reported	Oral health knowledge Oral health behaviours	This computer-based tool can be an effective way to deliver information to students about oral health in order to improve their oral health behaviours.
15. Hashemian et al. [46]	2015	United States	Randomised controlled trial	Mean age: 10 yrs. N = 156	The Intervention Group received text messages inquiring if they had used dental floss and providing oral health details for seven days. The regular printed materials from the clinic were also provided. The Control Group received the printed materials from the clinic only.	None reported	Oral health behaviour Oral health knowledge	Mothers receiving text messages demonstrated improvement in their own oral health behaviour and oral health knowledge and in the oral health behaviours of their children.
16. Yekaninejad et al. [47]	2012	Iran	Randomised controlled trial	Age: 11–12 yrs. Mean age: 11 N = 392 (1 = 131; 2 = 127; C = 134)	Schools were randomly assigned to three study groups: a comprehensive intervention group, a student intervention group, and a control group. The study assessed oral health using various measures including self-reported questionnaires, self-efficacy scales, oral health behaviour reports, and clinical examinations using the Oral Hygiene Index and the Community Periodontal Index. The student intervention group received three classroom sessions and homework over two weeks, delivered by a health education specialist. The comprehensive intervention group received the same student intervention and in addition, parents, school staff, and teachers were given a booklet about children’s oral health. This group also received booklets to take home. Students completed the questionnaires. The clinical examinations were conducted by two calibrated dentists at baseline, at two weeks, and at three months after the initial assessments.	Health Belief Model	Oral health behaviours (tooth brushing / flossing)Oral Health status: Oral hygiene index Periodontal Index	Promising results are seen when oral health education is targeted in both school and home settings.
17. Mohamadkhah et al. [48]	2013	Iran	Quasi-experimental study	Age: 10–12 yrs. N = 300	Students were divided into three groups: Control Group, Intervention Group 1, and Intervention Group 2. Intervention Group 1 watched an educational film, while Intervention Group 2 received a lecture about oral health education. All three groups underwent pre-tests. Thereafter, interventions were carried out in the Intervention groups; no changes were made in the Control Group. The study measured the effects of the educational oral health programme on health knowledge, attitudes, and practices immediately after the intervention and three months later.	None reported	Oral health promotion and knowledge Oral health behaviour (dental flossing)	The use of an educational film could be effective in promoting students’ self-care behaviours.
18. Nguyen et al. [49]	2016	Vietnam	Cross-sectional analytical	Age: 8–10 yrs. N = 556	The School Oral Health Promotion programme (SOHPP) was the intervention evaluated. Children’s knowledge of oral health and their self-reported oral health behaviours were assessed. Clinical examinations to assess the oral health status were then conducted by two calibrated dentists.	None reported	Oral health knowledge Oral health behaviour Oral health status: Dental caries	The prevalence of dental caries and gingivitis in Vietnamese children was high, and the School Oral Health Promotion Programme did not contribute towards improving their oral health behaviours.
19. Ghaffari et al. [23]	2018	Iran	Systematic Review	School children; Adults	Oral Health Education and Promotion Interventions (OHEPIs)		Oral health knowledge Oral health attitude Oral health behaviour Oral health status: Dental caries Gingival bleeding	Oral Health Education and Promotion Interventions were successful in bringing about favourable changes in dental visits, attitudes, and the habits of brushing and flossing among children in the three months following the intervention.
20. Xiang et al. [24]	2020	China	Systematic Review	Age: 10–19 years	Oral health behaviour interventions		Oral health knowledge Oral health attitude Oral health practices Oral health status: Plaque Index Gingival Index	Moderate evidence exists to support the effectiveness of behavioural interventions for oral health among adolescents. Future research needs a longer-term follow-up with detailed descriptions of the behaviour change intervention.
21. Wu et al. [50]	2017	China	Randomised controlled trial	Age: 12–13 years N = 512	The interventions evaluated included three groups viz: Oral health education Motivational interviewing Motivational interviewing and risk assessment with cariogram A calibrated examiner conducted the clinical examinations	Motivational interviewing	Oral health behaviour: Tooth brushing Diet Oral health status: Plaque index Dental caries (ICDAS)	Motivational interviewing was more effective than traditional oral health education in improving oral health behaviours and preventing dental caries in adolescents.
22. Xiang et al. [51]	2022	China	Cluster-randomized controlled trial	Age: 13 years N = 1184	The intervention group received a peer-led oral health intervention which included oral health talks, hands-on brushing workshops, posters, and oral health promotion leaflets. The control group only received oral health promotion leaflets.	Health Belief Model Social cognitive theory	Oral health behaviour; Tooth brushing Flossing habits Oral health status: Dental Caries Plaque index	Health Belief Model and Social Cognitive Theory in a peer-led intervention effectively improves adolescents’ self-reported brushing habits, oral hygiene status, and quality of life related to oral health
23. Swe et al. [52]	2021	Myanmar	Before and after paired	Age: 8–10 years N = 220	The intervention group received oral health education sessions using visual aids like charts and a dental model every eight weeks for a year, while the control group did not receive this education until after the study was completed.	None reported	Oral health-related knowledge, Oral health attitude Oral health practices	Oral health education, involving interactive talks, demonstrations, and supervised tooth brushing sessions every eight weeks over a year, effectively promoted and maintained accurate knowledge and behaviour among school children
24. Suresh et al. [53]	2023	India	Cluster-randomised control trial	Age: 6–12 years N = 453	Stage 1 involved a baseline survey and a clinical oral examination, both done by three trained dentists. Stage 2 included three oral health education (OHE) sessions delivered by a dentist and spaced one month apart for 3 months. Participants received OHE materials which included charts, pamphlets and a video were also used to convey the information. The control group did not receive any classes or educational materials.	Health Belief Model	Oral health-related knowledge, Oral health attitude Oral health practices Oral Health status: Plaque index Dental caries	The intervention successfully improved children’s oral hygiene and related knowledge, attitudes, and practices. However, additional long-term follow-up and economic evaluation are necessary
25. Nguyen et al. [54]	2021	Vietnam	Cluster -randomised control trial	Age: 12 years N = 462	The intervention included oral health education (OHE) facilitated by a dentist, utilizing a PowerPoint presentation along with hands-on tooth brushing and mouth observation. An oral health-related self-administered survey was conducted, and clinical examinations were performed at baseline and six months later by two calibrated dentists.	None reported	Oral health knowledge Oral health attitude Oral health behaviour Oral health status: Caries index Debris/plaque index	Participants in the intervention group demonstrated better oral health knowledge and behaviour compared to the control group. However, the intervention did not influence dental caries or gingivitis.

**Table 2:** Characteristics of included studies.

Characteristics	n (%)
**Study setting (N = 25)**	
School	21 (84)
Community	1 (4.0)
Combined (private practice and community)	1 (4.0)
Multiple settings (review articles)	2 (8.0)
**Study population (N = 23)** *	
Directly addressed (directed towards children or adolescents)	21 (91.3)
Indirectly addressed (directed towards parents and/or teachers)	2 (8.7)
**Study design (N = 25)**	
Systematic review	2 (8)
Randomised control trial	12 (48)
Quasi-experimental study	3 (12)
Before-and-after experimental (paired) study	6 (24)
Before-and-after experimental (unpaired) study	1 (16)
Cross-sectional analytical study	1 (16)
**Outcome measures (N = 23)**	
Combined (clinical and non-clinical)	14 (60.0)
Non-clinical	9 (39.1)
**Type of oral health intervention (N = 23)** *	
Oral health education via computer	1 (4.4)
Oral health education via text messages	1 (4.4)
Oral health education via small group card game	1 (4.4)
Oral health education via face-to-face presentation	9 (39.1)
Oral health education via face-to-face presentation and tooth-brushing activity	9 (39.1)
Motivational interviewing	2 (8.7)
**Continent of study (N = 23)** *	
Asia (includes Turkey)	15 (65.2)
Europe	5 (21.7)
Africa	2 (8.7)
North America	1 (4.3)
**Year of publication (N = 26)** ^#^	
2011–2012	3 (11.5)
2013–2014	4 (15.4)
2015–2016	6 (23)
2017–2018	4 (15.4)
2019–2020	5 (19.2)
2021–2022	4(15.4)
2023	0 (0)

^#^Number of included studies is 25 but number of publications is 26 as the study of Haleem et al. (2012) has two publications

*Excludes systematic reviews

Table 2 presents a summary of the characteristics of the included studies. Most studies were conducted in schools (84%). In most of the studies (91.3%), the interventions were directly conducted with children and adolescents and indirectly with those who had an influence on oral health behaviour such as parents, guardians, and teachers (8.7%).

One study that resulted in improving the children’s OHK used a card game as the medium to deliver oral-health-related information to schoolchildren [35], while another study directed at improving the oral-health-related knowledge and behaviours of mothers used mobile phones to deliver text messages to these mothers for repetition and reinforcement [44].

The clinical outcome measures refer to outcomes in which a clinical index was used to measure the outcome, while the non-clinical outcome refers to outcomes such as knowledge and self-reported behaviour.

**Fig 2:**
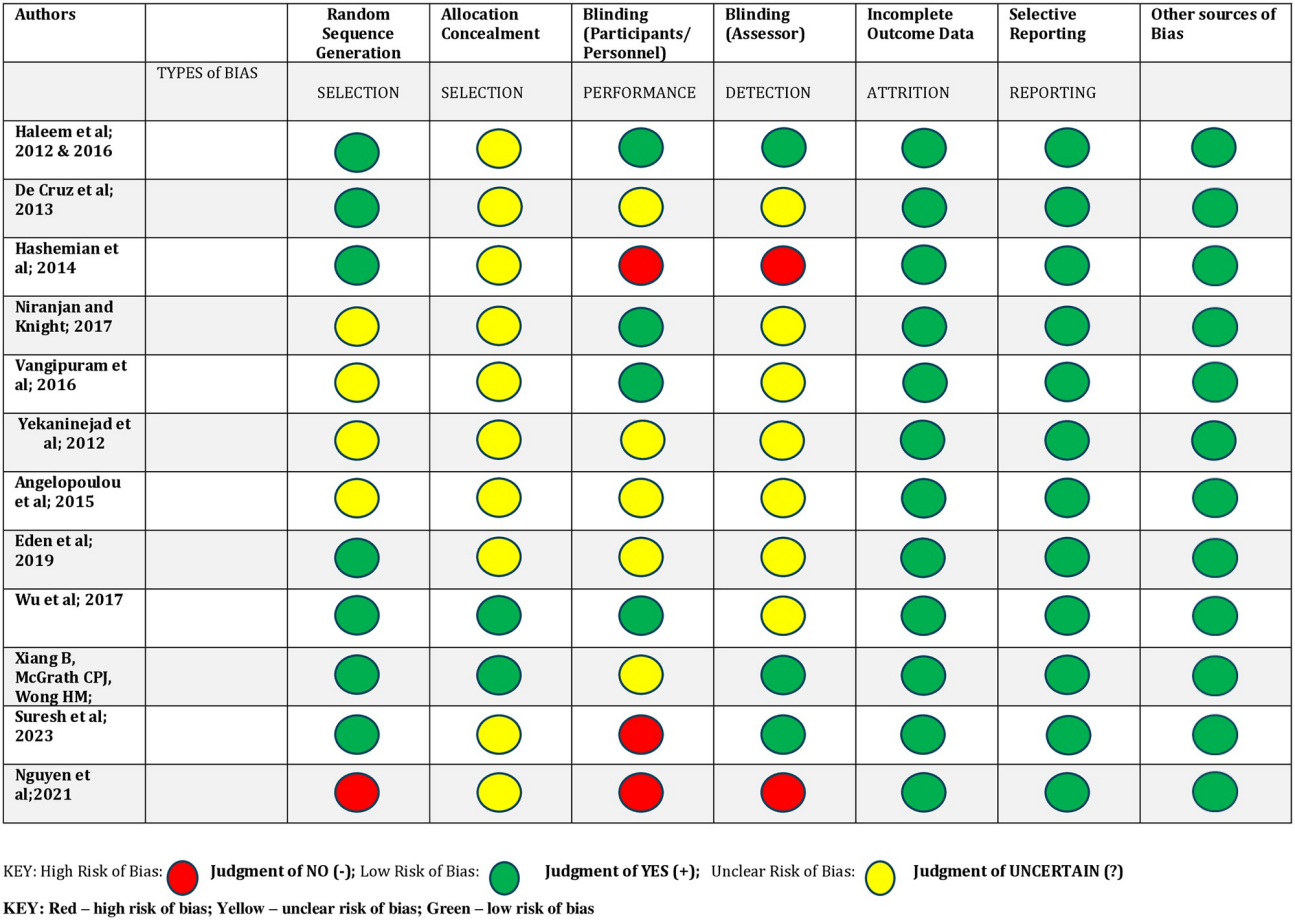
Risk of bias for the randomised controlled trials. An overall interpretation of the quality of the twelve RCTs is shown in Fig 2. None of the studies was completely free of the possibility of bias.

A RoB assessment (Fig 2) of the twelve randomised control trials (RCTs) was conducted to assess the quality of these studies. The other types of study designs were quasi-experimental, before-and-after experimental (paired and unpaired), and observational cross-sectional analytical (Table 2). Most of the articles included in this review were published between 2016 and 2022(Table 2) and were conducted primarily in Asia (65.2%), followed by Europe (21.7%).

Three studies had only one of the seven methodological items assessed as an unclear risk of bias. Of these 3 studies, 1 was not clear about allocation impacting on selection bias and the other 2 were unclear about blinding. Five studies had three of the seven items identified as problematic, and in one study, two items indicated a high risk of bias. Three studies had four of the seven items identified as having an unclear or high risk of bias. Also in one study, high risk of bias was reported for selection, performance, and detection bias, making it a poor-quality RCT. This indicates that in many cases, important methodological issues were not adequately reported, and thus the potential risk of bias must be considered when interpreting the findings.

In terms of this specific methodological issue, six trials provided detailed information on random sequence generation methods using the lottery method, a computerised processer, a table of random numbers, or computer-generated numbers [36, 37, 40, 41, 46, 50, 51]. However, for other RCTs, the sequence generation was unclear [34, 42, 44, 47, 54]. Only two RCTs indicated the method of allocation concealment and thus have low risk of bias [50, 51].

Regarding blinding, four studies were deemed to have a low RoB by blinding participants and personnel [40, 41, 47, 50]. One study did not blind the participants, personnel nor the assessor [54]. Another study blinded only the assessor [53]. Only one study achieved a low RoB by also blinding the assessors [36]. All studies had a low risk of bias in terms of presentation of outcome data and selective reporting, and no other sources of bias were detected.

#### Types of oral health interventions used during oral health promotion with children

Of the 23 individual studies, nine involved traditional OHE with face-to-face education, delivered via lectures (using PowerPoint or ‘chalk and talk’ methods) or videos (Tables 1 and 2). Three studies reported that OHE was delivered by alternative methods such as a computer program, SMS communication via mobile phone, or a card game (Tables 1 and 2). Nine studies included face-to-face OHE and a tooth brushing activity. Two studies reported using motivational interviewing (MI) (Tables 1 and 2). Of those that used OHE, information booklets were provided after the presentation. The people responsible for delivering the OHI varied and included dentists, dental hygienists, dental nurses, health education specialists, teachers, parents, or peers.

#### Effectiveness of oral health interventions

Effectiveness was measured through parameters such as the Plaque Index, Gingival Index, and Dental Caries Index (Decayed Missing and Filled Teeth [DMFT]) (Table 1) [33, 34, 38, 40, 47, 49, 50, 52–54]. Effective interventions included OHE with practical tooth-brushing activities, games, and audio-visual aids [31, 33, 36, 40, 42, 47, 51–54]. Motivational interviewing also proved to be an effective intervention [34, 50]. Moreover, the OHIs that used repetition and reinforcement after the initial intervention similarly improved outcomes [37].

#### Psychological theories underpinning effective oral health interventions

Only eight studies reported that the OHI was based on some form of a theoretical/conceptual framework or behaviour change theory (Table 1). A summary of the main characteristics of the frameworks or theories is presented in Table 3. The theories include the health belief model, the social cognitive theory (SCT), and the theory of planned behaviour. Table 3 does not contain MI since MI is a counselling strategy that was developed pragmatically and is based on practical experience rather than a psychological theory [55]. According to the RCTs with a low RoB, MI and the SCT and SCT combined with the HBM underlie effective interventions for modifying oral health behaviour in children [36, 50, 51].

**Table 3:** Characteristics of psychological theories underpinning reported studies.

Theory	Description	Core concepts/constructs	Notable theorists
**Health Belief Model** [56]	The Health Belief Model explains health behaviour in terms of an individual’s decision-making. The model proposes that the likelihood of a person adopting a health-related behaviour is related to four factors: the perceived threat; the balance between perceived benefits and the perceived barriers to these benefits; one’s self-efficacy; and finally, specific cues that prompt action. The perceived threat is related to the perceived seriousness of the problem and one’s perceived susceptibility to it. All these elements are subject to modifying factors in the person or in their environment.	Perceived susceptibility Perceived severity Perceived benefits Perceived barriers Cues to action Self-efficacy	Rosenstock (1952)
**Social cognitive theory** [57]	The social cognitive theory is a three-way reciprocal theory expanded from Bandura’s social learning theory. The social cognitive theory involves a dynamic interplay between a person’s personal factors, the person’s behaviour, and environmental influences. The social cognitive theory identifies humans as being capable of self-regulation and as having the ability to influence their environment and to be influenced by this environment. Cognitive factors play an important role together with environmental factors in the production and acquisition of new behaviours.	Human agency Observational learning (attention, retention, production, motivation) Triadic reciprocal determinism (cognitive, behavioural, and environmental factors) Self-efficacy	Bandura (1986)
**Theory of planned behaviour** (Ajzen as cited in Armitage and Connor [58]	The theory of reasoned action forms the basis of the theory of planned behaviour. The theory of reasoned action is applicable when a person’s voluntary behaviour is predicted by the person’s attitude towards the behaviour and how other people would view this person if the particular behaviour were performed. A person’s attitude combined with subjective norms forms their behavioural intention. The theory of planned behaviour was developed when the concept of self-efficacy was incorporated into the model.	Behavioural intention Perceived behavioural control (control beliefs and perceived behaviour) Attitude Subjective norms	Ajzen (1988)

## Discussion

### Summary of key findings

The OHIs used in 91% of the studies made use of traditional OHE where the primary difference between the studies was related to the medium used to deliver OHE and if a toothbrushing activity was included. Two studies reported using a different approach, namely MI [34, 50]. Approximately 39% of the studies only evaluated OHK (Table 2). Although these studies revealed an improvement in OHK, firm conclusions regarding effectiveness could not be reached. The studies that evaluated clinical outcomes suggest that employing OHE that is more participatory and engaging (e.g. with tooth-brushing activities, games, or audio-visual materials) together with MI is effective. Many of these clinical trials, however, had an unclear RoB and lacked long-term follow-up. According to two robust RCTs with a low RoB, MI and the SCT underlie successful interventions for improving oral health behaviour in children and could be considered to inform future OHIs. The included studies also imply that it is not necessary for a dentist to provide OHE, and this can be effectively done by [59] teachers or even peers [36]. Repetition and reinforcement of OHP has also been shown to improve adherence to behaviour change [37].

#### Discussion of key findings

Although traditional OHE contributes to an increase in OHK, this improvement is temporary and does not necessarily translate into improving clinical oral health outcomes and having a positive impact on the individual’s oral health status [22, 25].

Many of the studies measured OHK, and oral health behaviour was self-reported. Although the studies demonstrated that knowledge was acquired, this did not necessarily reflect a change in behaviour, particularly where no clinical outcomes were measured [59]. An improvement in oral health literacy may improve oral health outcomes [60, 61]. However, this association cannot be assumed, and it is preferable for clinical outcomes to be measured to confirm this. Fourteen studies in this ScR reported on clinical oral health outcomes. Another review concluded that there is a relationship between clinical oral health status and oral health-related quality of life (OHRQoL) [60].

In this review, school-based interventions were dominant despite evidence that the efficacy of primary school-based behavioural interventions is limited [28, 62]. The OHIs at primary schools mainly focused on the provision of traditional OHE and tooth-brushing demonstrations, and except for two RCTs that provided a detailed description of the OHI, reporting of the OHIs in the remaining studies was poor (Table 1) [36, 37]. There was a lack of standardisation in how the OHIs were reported and in fact, none of the OHIs utilised reporting guidelines for a behaviour change intervention. In addition, OHIs delivered in schools were poorly reported, making it difficult to identify the active components that were associated with the outcomes [20]. This highlights the need for OHIs to be reported using a comprehensive and standardised approach in order to allow for comparisons in systematic reviews and meta-analyses [50]. In addition, the heterogeneity of the studies and the poor reporting made a meta-analysis difficult [23]. Furthermore, important statistical values such as odds ratios and relative risk ratios (OR and RRR) were not available [23].

Although traditional OHE in schoolchildren reduces the levels of plaque in the short-term, there was a lack of long-term evidence regarding the effectiveness of school-based interventions in reducing plaque accumulation, gingivitis, and dental caries [28]. A systematic review of the OHIs included in this ScR concluded that the positive impact of the intervention on dental visits, attitudes, brushing, and flossing was demonstrated in the three-month follow-up after the intervention [23].

The use of digital media and games is reported as having some value and is gaining popularity in delivering oral health-related messages [63]. Digital media has a powerful impact on promoting good oral health across all age groups. Its widespread use in everyday life offers an excellent opportunity to influence oral health behaviours through OHE and OHP [63].

#### Theoretical frameworks

Theory-based approaches to health behaviour modification have been beneficial in other fields of medicine and could be used for OHP [64]. Other studies confirm that OHP underpinned by a psychological or behavioural theory is effective [14, 35, 36, 47].

A previous review of the effectiveness of school-based interventions concluded that none of the studies were underpinned by a psychological theory [25]. Another study explored the effectiveness of psychological interventions on the oral health of adolescents and adults and found no significant differences in gingivitis or plaque presence when comparing different interventions for periodontal disease [65]. However, one analysis showed a small but statistically significant difference in favour of psychological interventions for the Plaque Index [64]. Psychological interventions also had statistically significant benefits in terms of oral health behaviour and self-efficacy in tooth brushing, although the clinical significance of these findings is uncertain. Overall, the certainty of the evidence was considered low [65].

There is a need to apply psychological theory-based interventions not only to studies in adults with periodontitis but also to studies in adolescents with poor oral health [65]. Until October 2012, there was a lack of theory underpinning the OHIs [25, 26]. However, since 2012, an increasing number of OHIs are reported to be underpinned by a psychological theory [24, 34, 37, 39, 43, 47, 51, 53, 64, 66].

There is limited evidence for the effectiveness of OHIs in reducing tooth decay and plaque levels [25]. Furthermore, the interventions do not appear to be grounded in behavioural theory [25]. Additional high-quality research that incorporates behavioural theory is needed to improve interventions for changing oral health behaviours in children and parents [25].

This ScR identified eight publications that reported OHIs with a theoretical and/or conceptual framework. There was, however, a lack of detail regarding how the theoretical framework informed the intervention and, in some studies, it appeared as if the theory informed the questionnaires rather than the design of the intervention [39, 47]. None of the studies with an underlying psychological theory included in this ScR reported using a reporting guideline for the behaviour change intervention [36, 37, 39, 43, 47, 50].

Four RCTs in this ScR reported using psychological theories [36, 45, 51, 53]. The two publications of Haleem et al. demonstrated different objectives [36, 37]. However, the psychological theories used by Haleem et al. in their 2012 and 2016 articles were conflicting as the one article reported using the SCT [36] and the second cited the use of the social learning theory [37]. Following a personal communication, the first author, Haleem, confirmed that there was an error in the article published in 2016 and mentioned that it was in fact, the SCT and not the SLT that was used in the article published in 2016 (personal communication, Haleem, 2016, 5 December 2022). Although the study reported using the constructs of the SCT by including interactive group activities, details of how this theory informed the rest of the intervention were not clear [37].

A practical counselling strategy for behaviour change reported on in recent times is MI [34, 50]. Motivational interviewing is a person-centred collaborative form of counselling that involves using an individual’s intrinsic motivation to encourage the person to participate actively in changing their behaviour [67]. Motivational interviewing focuses on exploring and resolving ambivalence in addition to eliciting change talk [67]. There is an overlap between the concepts of MI and the self-determination theory, where these two counselling perspectives can be considered as being complementary [55]. The effectiveness of MI in healthcare is connected to self-determination [55]. The emphasis of autonomy support and goal-directedness between MI and the self-determination theory are however different [55].

Two studies were reviewed that used MI; in the first study [34], MI was directed at parents and in the second study [50], at adolescents. The first MI study included in this ScR reported that a single MI session positively contributed to changing the oral health behaviour of parents when compared with traditional OHE [34]. However, the study did not provide exact details of the MI intervention except to say that “a 30 min counselling session using a modified and translated protocol” was used [[34] p.193]. Further investigation revealed that the protocol was based on earlier work by Weinstein [unpublished] and incorporated the original principles of MI (personal communication, Weinstein, 11 October 2023).

The second MI study with a low RoB reported on a detailed MI intervention with positive results [50]. This MI study was a single-blinded RCT that investigated the effectiveness of MI in improving the oral health of adolescents [50]. The study provided a detailed description of the MI intervention and tested the fidelity of the MI intervention using the Motivational Interviewing Treatment Index tool [50]. The study also measured behavioural, clinical, and self-reported outcomes and found that the MI intervention was more effective than traditional OHE in improving adolescents’ oral health behaviours and preventing dental caries [50].

## Limitations

The limitation of the ScR was that it was restricted to articles published in English and thus may have missed useful evidence in other languages. In addition, grey literature such as conference proceedings and unpublished theses and studies was not sought. Furthermore, the search was limited to specific databases, and relevant studies might have been contained in other databases.

## Implications

The findings of this ScR can inform the design of future OHIs in children. Key points to consider include the following:

1. A person-centred approach such as MI
2. Social cognitive theory and/or health belief model
3. A practical tooth-brushing activity such as demonstration and/or supervised tooth brushing
4. The use of games and audio-visual aids
5. Repetition and reinforcement through the medium of either text messages or telephone calls to improve patient compliance

Overall, there is a need for additional high-quality studies to be conducted that provide a detailed description of the behaviour change intervention, including behaviour change reinforcements. Furthermore, future studies should focus on longer-term follow-ups and the quality control of the intervention.

## Conclusions

Most of the OHIs investigated in this ScR primarily focused on using traditional OHE in various forms and delivery methods. While these interventions led to short-term improvements in OHK, their effectiveness in changing oral health behaviour and clinical outcomes remains inconclusive. The quality of reporting and standardisation of OHIs in schools was often poor, making it challenging to identify active components associated with positive outcomes.

Successful approaches incorporate motivational interviewing (MI), social cognitive theory, health belief model, practical tooth brushing techniques, gamification, and audio-visual methods. Additionally, strategies benefit from consistent reinforcement and repetition.

This review emphasizes the need for higher quality studies on oral health interventions for children. Future research should include more detailed descriptions of interventions and longer follow-up periods.
